# *In ovo* uptake, metabolism, and tissue-specific distribution of chiral PCBs and PBDEs in developing chicken embryos

**DOI:** 10.1038/srep36597

**Published:** 2016-11-07

**Authors:** Zong-Rui Li, Xiao-Jun Luo, Li-Qian Huang, Bi-Xian Mai

**Affiliations:** 1 State Key Laboratory of Organic Geochemistry and Guangdong Key Laboratory of Environmental Protection and Resources Utilization, Guangzhou Institute of Geochemistry, Chinese Academy of Sciences, Guangzhou 510640, China; 2 University of Chinese Academy of Sciences, Beijing 100049, China

## Abstract

Fertilized chicken eggs were injected with environmental doses of 4 chiral polychlorinated biphenyls (PCBs) and 8 polybrominated biphenyl ethers (PBDEs) to investigate their uptake, metabolism in the embryo, and distribution in the neonate chicken. PCB95 uptake was the most efficient (80%) whereas BDE209 was the least (56%). Embryos metabolized approximately 52% of the PCBs absorbed. Though some degree of metabolism in the first 18 days, most of the PCBs and PBDEs was metabolized in the last three days, when BDE85, 99, 153, and 209 decrease by 11–37%. Enantioselective metabolism of the (+) enantiomers of PCB95, 149, and 132 and the (−) enantiomer of PCB91 was observed. The enantioselective reactivity was higher with the two penta-PCBs than the two tetra-PCBs. Liver, exhibited high affinity for high lipophilic chemicals, enrich all chemicals that was deflected in other tissues except for some special chemicals in a given tissues. Lipid composition, time of organ formation, and metabolism contribute to the distribution of chemicals in the neonate chicken. The result of this study will improve our understanding on the fate and potential adverse effects of PCBs and PBDEs in the neonate chicken.

Polychlorinated biphenyls (PCBs) and polybrominated diphenyl ethers (PBDEs) have raised worldwide concern due to their persistence, long-range transport, bioaccumulation, and toxicity. These ubiquitous environmental pollutants have been found at low but measurable levels in nearly all animals and humans^1^,^2^. Their adverse effects include perturbations of the immune, reproductive, nervous, and endocrine systems; they are also known to have oncogenic effects^3^,^4^. The persistent pollutants such as PCBs and PBDEs can enter into egg by maternal transfer and cause adverse effects on the embryo, given that early development stages are among the most vulnerable periods in the life cycle. de Roode *et al.*^5^ observed embryos with malformation after exposure to fraction associated with the most persistent organic pollutants, such as PCB, polychlorinated diphenyl ethers, PBDEs, and organochlorine pesticides which extracted from guillemot eggs from Baltic Sea and Atlantic Oceas. However, there has been relatively little experimental work on the effects of this maternal transfer of contaminants to embryo development and few studies were conducted to investigate the behavior and fate of PCBs and PBDEs during the embryo development.

PCB and PBDE toxicities vary with congener, target species, and animal age, and with the enantiomer in the case of chiral PCBs^6^,^7^,^8^. Individual PCB enantiomers may interact enantioselectively (or enantiospecifically) with chiral macromolecules, such as cytochrome P-450 enzymes or ryanodine receptors, leading to differences in their toxicological effects and the enantioselective formation of chiral biotransformation products^9^. Avian embryos are considered an ideal animal model for studying the developmental toxicity of xenobiotics. They are relatively large and the chick develops in an egg ex utero, which allows easy accessibility and manipulation during all stages of post-laying development. Moreover, the chicken is very sensitive to the embryotoxic effect of xenobiotics such as PCBs and PBDEs^10^,^11^.

Contaminants have been injected into the air cell or yolk sac to assess their direct effects on embryo development. This technique allows for the use of specific amounts of xenobiotics^10^,^12^. In previous studies, de Roode *et al.*^13^ reported PCB uptake from the yolk of the developing chicken embryo after Aroclor 1254 (a commercial mixture of PCBs) injection. McKernan *et al.*^12^ studied PDBE absorption and biotransformation in the eggs of several avian species following air cell administration of penta-PBDE and octa-PBDE mixtures. Winter *et al.*^14^ validated the use of egg injection to study *in ovo* xenobiotic exposures by the injection of PBDE99 directly into the yolk sacs of model songbird eggs. Nevertheless, the existing studies focus mainly on absorption, not on biotransformation or tissue distribution in early life stages. Moreover, little is known so far about the enantioselective metabolism and tissue distribution of chiral PCBs even though chiral PCB alternation has been reported in many studies on the adult avian^15^,^16^,^17^.

In the present study, fertilized chicken eggs were collected from an unpolluted area and exposed by injection to certain doses of contaminants. The present study aimed to 1) explore the uptake efficiency, 2) examine the metabolic capability, and 3) investigate the tissue-specific distribution of four chiral PCBs and eight PBDE congeners in developing chicken embryos. Through this research, we hope to elucidate the biotransformation and tissue distribution of organohalogenated compounds in the early life stages of terrestrial avians.

## Results and Discussion

### Uptake of PCBs and PBDEs during chicken embryo development

The mass of four chiral PCBs and eight PBDE congeners in eggs, neonate tissues (liver, heart, stomach, and carcass) and the remaining yolk are presented in Table 1. The difference in chemical mass between the day-0 egg and the remaining yolk was taken as the uptake mass during embryo development. The uptake efficiencies of 12 target chemicals ranged from 57.5 ± 0.03% (standard error, BDE209) to 80.0 ± 0.03% (PCB95) (Supplementary Fig. S1). No significant differences in uptake efficiencies were found among the chemicals except for PCB95 and BDE209 (one-way ANOVA with post-hoc test). The uptake efficiency of BDE209 was 56%, significantly lower than those of the other chemicals but not negligible. BDE209 has a larger molecular volume than the other compounds, which may account for its relatively low uptake efficiency. It is thought that BDE209 is less readily transferred than the other chemicals because of its relatively high molecular mass. In a previous study^12^, where DE-79 (octa-BDE mixture contains 5 percent of BDE209) was injected into the air cells of eggs, BDE209 was not detected in developing embryo. The egg membrane may have retarded the diffusion of BDE209 into the egg.

De Roode and van den Brink^13^ reported that 18% of the injected PCBs was absorbed 19 days after incubation. In the present study, 70–80% of PCB congeners were absorbed. The uptake increased exponentially with the incubation period. Therefore, the rapid absorption in the last 3 days might contribute to the high uptake efficiency observed in this study. Additionally, metabolism of PCBs in developing embryo can reduce uptake efficiency. Only a small proportion of PCBs was thought to be metabolized according to the study of De Roode and van den Brink^13^. In the present study, however, up to 17% of the original PCB mass was metabolized within the first 18 days of incubation. Therefore, the actual uptake of PCBs in the embryos might be underestimated by De Roode and van den Brink^13^.

### Metabolism of PCBs and PBDEs during chicken embryo development

After 18 days of incubation, the PBDE content in the egg was almost the same as that of the day-0 egg (Table 1), indicating that no metabolism occurred. This finding is similar to that of our previous study^18^. Recent research, however, showed debromination in mid-incubation of bird embryos^12^. Thus, different chemicals may not be metabolized in the same way. The PCB content in day 18 eggs decreased relative to that in day-0 eggs. During the 18-day incubation, approximately 17% of PCB95, 8% of PCB132, 5% of PCB91, and 4% of PCB149 were metabolized by the embryos. Chicken embryos can metabolize xenobiotics like PCBs and PAHs according to previous studies^19^. Different degrees of metabolism among the four PCB congeners indicates chemical-specific metabolism during embryo development. This response has been demonstrated in adult chicken^20^,^21^.

**Figure 1:**
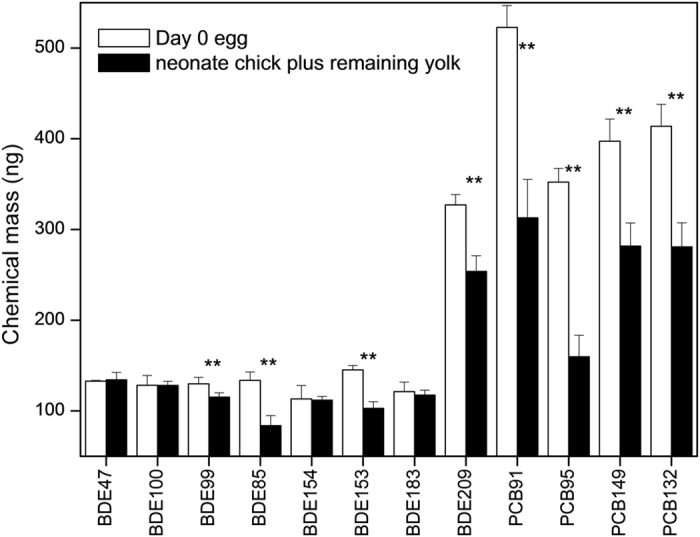
Mass balance (mean) of exposed chemicals between day-0 egg and neonate chick plus remaining yolk. Results are expressed as ng/egg or ng/chick. The double-asterisks represent significant difference (p < 0.05) and error bars represent standard deviations.

At the end of incubation, the mass of four PCB congeners in the neonate chick (including the remaining yolk) significantly decreased by 40%, 55%, 29%, and 32% for PCB91, PCB95, PCB149, and PCB132, respectively (Fig. 1). Based on the amounts of PCBs absorbed by the embryo, it metabolized between 40% (PCB149) and 69% (PCB95). Therefore, about half (52%) of the PCB absorbed by the embryo was metabolized. The mass of four PBDE congeners (BDE47, BDE100, BDE154, and BDE183) was not significantly different between neonate chicks and day-0 eggs. The mass of the other four congeners (BDE99, BDE85, BDE153, and BDE209) decreased significantly in neonate chicks compared with day-0 eggs (Fig. 1). The elimination rates were 16%, 50%, 40%, and 39% of the absorbed BDE99, BDE85, BDE153, and BDE209, respectively. The metabolism of PBDEs occurred only in the last three days of development. For the first two weeks of its development, the chick embryo relies mainly on carbohydrate and protein metabolism for its energy needs. The remaining seven days, and especially the last three days, are periods of intense lipid metabolism and rapid development. Up to 80% of the lipid content of the yolk is assimilated into the embryonic tissues at this time^22^,^23^, which also means high uptake and metabolic rates for PCBs and PBDEs.

PCBs with a 2,5-dichloro- or 2,3,6-trichloro substitution on one of their phenyl rings are preferentially metabolized to persistent methylsulfone metabolites^24^,^25^. All 4 PCB congeners used in the present study contain 2,3,6-trichloro-substituted phenyl rings. PCB95 contains both 2,5-dichloro- and 2,3,6-trichloro-substituted phenyl rings. The high metabolic rate of PCB95 may be related to its structure. Methylsulfone metabolites were not detected in this study, so there is no direct evidence to support the sulfomethylation metabolic pathway of PCBs. Maervo *et al.*^26^ suggest that both CB52 and CB101, which contain at least one 2,5-dichloro-substituted ring, were partially transformed in broiler chickens either to hydroxylated metabolites or conjugated compounds since no methylsulfone metabolites were detected. In birds, congeners with vicinal hydrogen atoms in the meta/para-position are more readily metabolized than those with a para-chlorine-substituted phenyl ring and adjacent unchlorinated ortho/meta-positions. Thus, CB95 is eliminated much faster than CB91.

Several studies reported that PBDEs could be metabolized in birds and chicken^12^,^14^,^18^,^27^,^28^. McKernan *et al.*^12^ found that six debromination congeners and two methoxylated metabolites in mid-incubation- and piping bird embryos injected *in ovo* with DE71. DE71 metabolites only appeared in the egg contents toward the end of incubation and in the piping chick.

It has been reported that BDE209 degraded to lower brominated congeners in European starlings^27^. In the present study, three nona-PBDE congeners were detected in the neonate chick. Nevertheless, they were also detected in day-0 eggs, and the ratio of nona-BDE to deca-BDE was not significantly different between day-0 eggs and neonate chicks (data not shown). Debromination may not be the main reason why BDE209 decreased in the neonate chick. It was reported that BDE209 is more rapidly eliminated than the low-brominated PBDEs^29^,^30^. The present study confirms this conclusion. As shown in Table 1, very few low-brominated PBDEs were detected in the remaining shell after piping. However, an average of 47 ng BDE209 was detected in the shell, which means that most of the adsorbed BDE209 was excreted.

It was found that BDE153 is the major congener in some field bird samples^31^,^32^. It is therefore puzzling that the amount of BDE153 decreased in neonate chicks relative to that in day-0 eggs. In female mice, BDE153 can be biotransformed into six mono-OH-BDEs^33^. It is still unclear whether BDE153 biotransformation occurs in birds. The nominal mass of BDE153 was the same as those of the other BDE congeners, but the mass of BDE153 measured in the day-0 egg was 15–20 ng higher than the other congeners (Table 1). It is therefore possible that a false positive was obtained for BDE153 metabolism in the present study.

Our research group conducted an *in vitro* metabolism study of BDE99, BDE47^34^ and BDE85 using chicken liver microsomes (unpublished). BDE47, BDE99, and BDE85 can be metabolized into at least two hydroxylated metabolites. We examined the potential OH-metabolites in neonate chicks by partitioning the extract with a potassium hydroxide solution (see Supplementary Method) and comparing it with the *in vitro* experiment using BDE85. A metabolite of BDE85 was confirmed in the neonate chicken (Supplementary Fig. S2). Several potential metabolites were also found but it was difficult to identify them due to the lack of reference standards. Decreases in BDE99 and BDE85 levels can therefore be attributed to biotransformation. BDE47 levels in the neonate chicks and the day-0 eggs were similar. In an embryo development study of the zebra finch^14^, BDE99 debrominated to BDE47, which might explain why there was no significant change in BDE47 levels.

The structure-activity relationship of PBDE metabolism in biota is not clear yet. If the structure-activity relation of PCB metabolism can also be applied to PBDE, BDE100, BDE154, and BDE183 will be very difficult to metabolize since they lack vicinal hydrogen atoms in either the meta/para- or ortho/meta-position. This fact is corroborated by the findings of the present study.

### Enantioselective transformation of PCB atropisomer during embryo development

**Figure 2:**
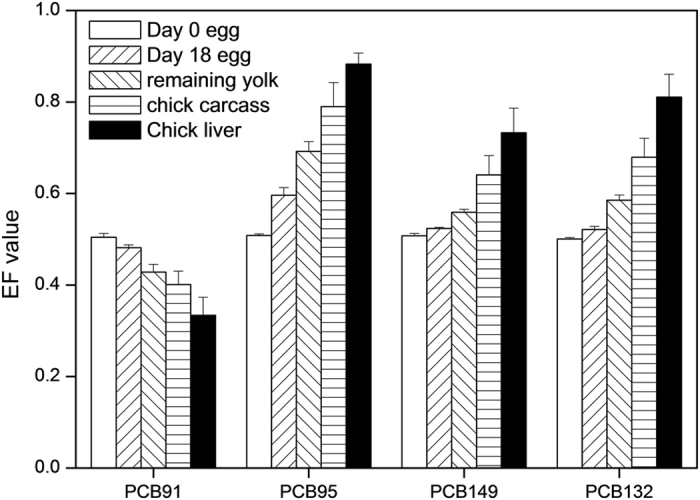
Enantiomer fractions of chiral PCBs (PCB91, 95, 149, and 132) in eggs (day 0 and day 18) and neonate chick tissues (remaining yolk, carcass, and liver). Error bars represent standard deviations.

To elucidate the enantioselective metabolism of PCB atropisomer during embryo development, the enantiomer fractions (EF) of four chiral PCBs were measured. The EF of PCB91, 95, 149, and 132 in day-0 eggs were 0.504 ± 0.008, 0.508 ± 0.004, 0.507 ± 0.005, and 0.500 ± 0.003, respectively (Fig. 2), indicating racemic. The EF values were 0.482 ± 0.006, 0.596 ± 0.017, 0.524 ± 0.003, and 0.521 ± 0.007, respectively in day 18 eggs. The extent of EF derived from racemic is consistent with that of metabolism shown above and confirms that PCB biotransformation occurs during the first 18 days of incubation.

Compared to day 18 eggs, the neonate chicks exhibited further atropisomeric enrichment for all four chiral PCBs, especially in the liver tissue. The EF values of the 4 PCBs in the remaining yolks lay between those of the day 18 eggs and the neonate chicks. The yolk is merely cytoplasm containing nutritional reserves for the developing embryo; biotransformation was not expected to occur there. Therefore, the changes in EF for the PCB in the yolk must originate from the neonate chick, and the material exchange between the developing embryo and the yolk must be bidirectional.

The concentrations of PCBs in the neonate chick heart and stomach were too low for EF measurement. Thus, only liver- and carcass EFs were obtained. The degree of EF divergence from 0.5 was greater in the liver than in the carcass since biotransformation occurred mainly in the liver. Similar results were found in adult hens raised in an e-waste recycling area^35^. Our previous study showed chemical specificity of the metabolic enantioselectivity for chiral PCBs. Enrichment of the (+) atropisomer was observed in the neonate for PCB95, PCB132 and PCB149 but (−) atropisomer enrichment was found for PCB135 in the chick. In the present study, selective biotransformation of the (+) atropisomer of PCB91 occurred, but (−) atropisomers of PCB95, PCB132, and PCB149 were also detected. This finding supports chemical-specific metabolic enantioselectivity for chiral PCBs. A possible explanation is that different cytochrome P450 (CYP) enzymes are degrading different chemicals.

The masses of (+) or (−) atropisomers metabolized by the neonate chick were determined. The EF values in the heart and stomach was though as same as the carcass, this will not introduce a large error due to the small amounts of PCBs in these two tissues. Fifty percent, 32%, 13%, and 12% of the (+) atropisomers of PCB91, PCB95, PCB149 and PCB132, respectively were metabolized. On the other hand, the percentages of (−) atropisomers metabolized were 30%, 77%, 45%.and 51%, respectively for the aforementioned PCBs. The ratios of (+) to (−) atropisomers metabolized can reflect relative differences between them in reactivity. The ratios were 1.7, 2.4, 3.5, and 4.3 for PCB91, PCB95, PCB149, and PCB132, respectively. The values were higher for the two penta-chlorinated congeners than for the two tetra-chlorinated congeners. Since there are only two data groups, we cannot confidently conclude that the more chlorine substitution in the phenyl ring, the greater the difference in reactivity between the two atropisomers. Further research is required.

### Tissue distribution of PCBs and PBDEs in the neonate chick

**Figure 3:**
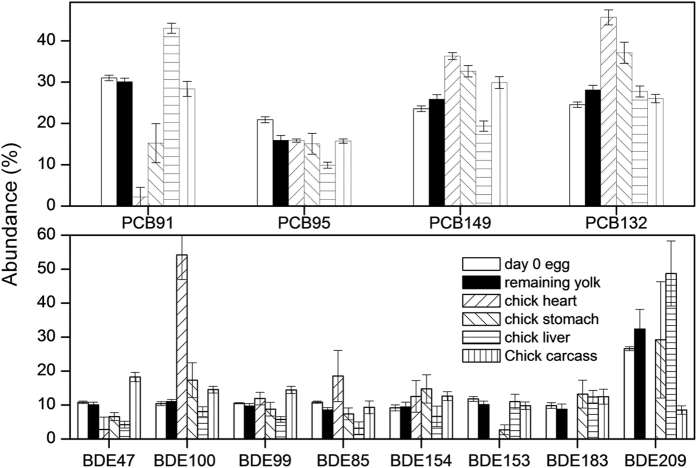
Profile of PCB and PBDE congeners in day-0 eggs and neonate chick tissues (remaining yolk, heart, stomach, liver and carcass). Error bars represent standard deviations.

The chemical composition pattern was examined to determine the affinity of the chemicals for the tissues; the composition pattern in the day-0 egg was used as benchmark. As can be seen from Fig. 3, different tissues have different chemical affinities, such as BDE100, PCB149 and PCB132 for the heart; BDE209 and PCB 91 for the liver; BDE47 and 99 for the chicken carcass; and BDE154 and 183 for the stomach. This information aids in understanding the fate and potential adverse effects of chemicals in the neonate chick.

There are few studies concerning chemical distributions in chicken embryo tissues. In one study, no differences were found in PBDE congener patterns among the various tissues of a certain raptor species^36^. The partition between blood and tissue is thought to be a determinant in the loading of chemicals in adult bird tissues. In bird embryos, however, different nutrients are used to form different tissues and organs during development^18^ and the times when the organs are formed also differ. The induction of metabolism is also time-dependent. The tissue distribution of chemicals in the embryo differs from that in the adult bird.

**Figure 4:**
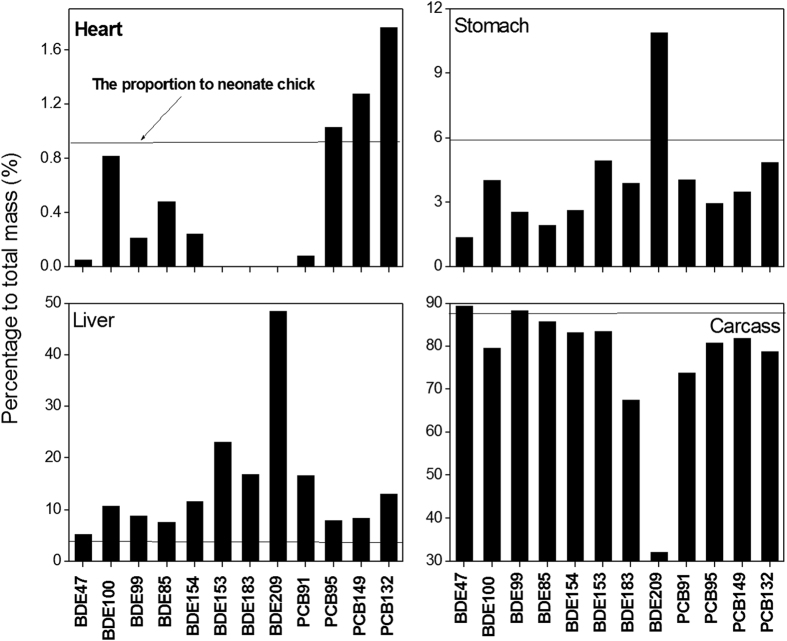
Percentage of chemicals (%) in each tissue relative to total mass of contaminant in neonate chick. The flat solid line represents the mass ratio of individual tissue to total neonate chick.

To determine whether a tissue enriches or deflect chemicals, a comparison the ratio of tissue weight to total chick weight with the ratio of tissue chemical burden to total chick chemical burden (Fig. 4) was conducted. If the latter was larger than the former, it means enrichment occurred in the tissue and vice versa.

**Figure 5:**
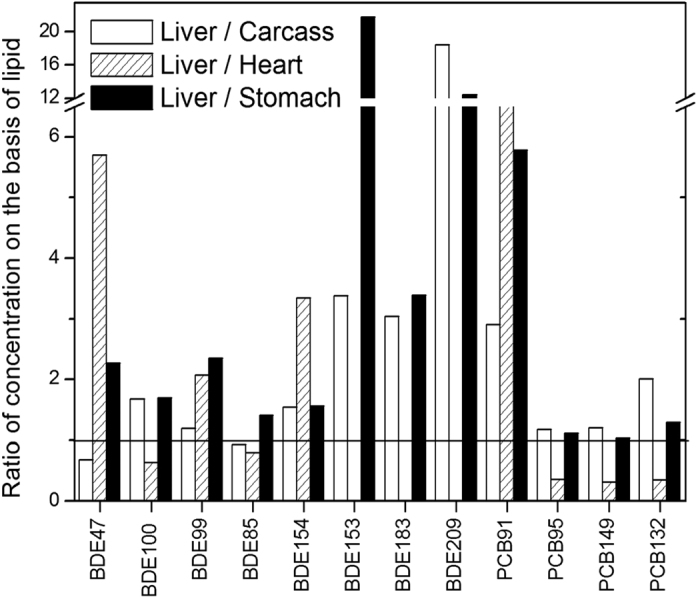
Ratio (liver/carcass, liver/heart, and liver/stomach) of concentration (ng/g lipid-weight) of individual chemicals. The flat solid line represents ratio = 1:1.

Enrichment of all chemicals was observed in the liver. The enrichment factor (i.e., the ratio of chemical percentage to mass percentage) ranged from 1.2 for BDE47 to 11.2 for BDE209. For chick carcass, all chemicals show defect except for BDE47 which show somewhat enrichment. The chick stomach enriched BDE209 and defect all other chemicals. The chick heart enriched PCB95, 149, and 132, and defect all other chemicals. Evidently the liver is the main target organ for chemical accumulation. The lipid content of the liver is the main (but not the only) reason why it accumulates chemicals. As seen in Table 1, the lipid content of the liver is twice that of the carcass and about 5–6 times that of the heart and stomach. Since all of the chemicals tested are lipophilic, high loading in the liver occurs. The lipid-normalized concentrations for most of chemical were still higher in liver than in other tissues (Fig. 5), indicating a higher chemical affinity in the liver. By the final week of embryo development, the lipid in the liver consists mainly of cholesterol ester. The major lipid fractions of the embryonic heart and skeletal muscle, however, are composed of phospholipid and free cholesterol^22^. PCBs and PBDEs have a much higher affinity for neutral lipid than for phospholipids^37^. This fact may account for the high levels of chemicals in the liver. The levels of BDE100, and 85, and PCB95, 149, and 132 were higher in the heart than the liver possibly due to metabolism in liver and the higher diffusion rate of these chemicals since heart is the first organ formed during embryo development. A positive correlation was found between the ratio of liver to carcass and the log *K*_*OW*_ of the chemicals (Supplementary Fig. S3), indicating that the liver tends to accumulate highly lipophilic chemicals. This result is consistent with our previous study wherein the ratio of muscle to muscle plus liver negatively correlated with log *K*_*OW*_. An exception was PCB91, perhaps because of its high liver affinity.

## Methods

### Exposure

Chicken eggs (*Gallus domesticus*, n = 25) were collected in August 2015 from a farmer in Qianjiang County, Hubei Province, China. All eggs were washed with 75% v/v aqueous ethanol solution then weighed and labeled before injection. Three eggs were sampled to determine background contaminant levels. The background concentrations of PCBs and PBDEs were negligible compared to the exposure group (Table 1). Following the egg injection protocol validated in a previous study^14^, dimethyl sulfoxide (DMSO, CAS 67-68-5) was used in the present study as the vehicle for the contaminants due to its low toxicity to developing embryos. The injection solution was prepared as follows: four chiral PCBs (PCB95, 91, 149, and 132) and eight PBDEs (PBDE47, 85, 100, 99, 154, 153, 183, and 209) standards were prepared, concentrated to near dryness under a gentle nitrogen flow, then dissolved in DMSO at a dose of about 5 μg/mL for all PBDE congeners except BDE209, and 20 μg/mL for PCB congeners and BDE209. These concentrations were selected on the basis of contaminant levels found in birds living in a polluted environment. Eggs were held upright with the pointed end (no air cell) down and the round end was cleaned with the 75% ethanol solution. A hole was made in the round end using a sterile needle and a syringe was pushed through the shell and into the egg for about 3 cm to reach the yolk. A constant volume (20 μL/egg) of the prepared solution was then injected into the yolk. After a few hours, 4 day-0 eggs were sampled to determine the exposure doses of individual congeners. The rest of the injected eggs were placed in an incubator for hatching. After the 4^th^ day of incubation, the eggs were candled to confirm their viability. Unfertilized or dead eggs were removed. Four eggs were randomly sampled on day 18. All samples were reweighed to determine weight loss. In our previous study, no significant changes in the enantiomeric fractions (EFs) of chiral PCBs were observed after 14 days incubation. Since albumen is the main source of nutrition for embryos in the first 18 days, followed by the yolk thereafter, day 18 was set as a sampling point. Six chicks were hatched then euthanized with nitrogen. Chicks were dissected and their hearts, livers, stomachs and their remaining yolk excised. Tissue samples were stored at −20 °C until further analysis. The study was approved by the Ethics Committee in the Guangzhou Institute of Geochemistry, Chinese Academy of Science and all methods were performed in accordance with the relevant guidelines and regulations.

### Sample preparation and analysis

Samples were extracted and cleaned according to a previously published method^38^. Approximately 2 g (dry weight) of the egg samples and all of the chick tissue samples were lyophilized then homogenized. Soxhlet extraction was performed on the samples for 48 h using 200 mL hexane/dichloromethane (1:1 v/v) spiked with surrogate standards (PCB24, 82, and 198; BDE118, and 128; 4-F-BDE67; 3-F-BDE153, and ^13^C-BDE209). The extract was concentrated to 10 mL by rotary evaporation, then a 1-mL aliquot was drawn off to determine the lipid content by gravimetric analysis. The remaining extract was treated with concentrated sulfuric acid (10 mL) for lipid removal then further purified in a multilayer gel column (length, 30 cm; inner diameter, 1 cm) filled from bottom to top with 20 cm Florisil, 2 cm neutral silica, 5 cm acid silica and 3 cm anhydrous sodium sulfate. The extract was eluted with 70 mL hexane followed by 60 mL dichloromethane. The first fractions containing PCBs and PBDEs were concentrated to near dryness under a gentle nitrogen flow then reconstituted in 200 μL isooctane for analysis. The extract was spiked with known amounts of the recovery standards (PCB30, 65, and 204; BDE77, 181, 205) before instrumental analysis.

Four chiral PCBs were analyzed by an Agilent 7890 gas chromatograph coupled to an Agilent 5975 mass spectrometer (GC-MS) using electron ionization in the selected ion monitoring mode. A DB-5MS capillary column (60 m × 0.25 mm i.d.; 0.25-μm film thickness) was used to separate the PCB congeners. The enantiomer signatures of the chiral PCBs were determined on a Chirasil-Dex column (25 m × 0.25 mm i.d.; 0.25 μm film thickness). The enantiomer fractions (EFs) were calculated by dividing the area of the (+)-enantiomer by the areas of both (+) and (−) enantiomers.

Eight PBDE congeners were quantified by an Agilent 6890 gas chromatograph equipped with an Agilent 5975 mass spectrometer in the electron capture negative ionization mode. Seven PBDE congeners (except for BDE209) were separated by a DB-XLB capillary column (30 m × 0.25 mm i.d.; 0.25 mm) and a DB-5HT capillary column (15 m × 0.25 mm i.d.; 0.10 mm) was used for BDE209. The details for the instrumental analyses of PCBs and PBDEs were described previously^38^.

### Quality assurance and quality control

Procedural blanks were run periodically for each batch of 9 samples. Trace amounts of the target chemicals were detected but the levels were <1% of the analyzed concentration in most samples. The recoveries were 76–101% and 88–106%, respectively, in the spiked blanks of 19 PCB congeners (PCB8-206) and 8 PBDE congeners (BDE28, 47, 100, 99, 154, 153, 183, and 209), with relative standard deviations (RSDs) <15% (n = 3) for all target chemicals. The recoveries of the surrogate standards were 105 ± 10%, 85 ± 9%, and 86 ± 7% for PCBs 24, 82, and 198, and 93 ± 7%, 88 ± 8%, 70 ± 17%, 89 ± 14%, and 97 ± 18% for 4-F-BDE67, BDE118, 3-F-BDE153, BDE128, and ^13^C-BDE209, respectively. The limits of quantification were set as a signal-to-noise ratio of 10 and ranged from 0.007–0.03 ng/g dw (dry weight) and 0.015–0.024 ng/g dw for PCBs and PBDEs, respectively.

### Statistical analysis

Statistical analyses were performed with SPSS V. 16.0 for Windows. The level of significance was set at *p* = 0.05 throughout the study. One-way analysis of variance (ANOVA) tests were used to determine the differences in contaminant levels and enantiomer fractions among sample groups.

Chemicals detected in the remaining yolk were treated as fractions of the injected dose not absorbed during embryo development. We calculated the uptake efficiency and metabolic rate as follows:


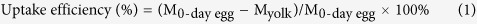



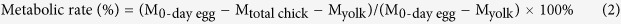


where M_0-day egg_, M_yolk_ and M_total chick_ represent the mass of contaminant in the day-0 egg, the remaining yolk and the neonate chick, respectively.

## Additional Information

**How to cite this article**: Li, Z.-R. *et al.* *In ovo* uptake, metabolism, and tissue-specific distribution of chiral PCBs and PBDEs in developing chicken embryos. *Sci. Rep.* **6**, 36597; doi: 10.1038/srep36597 (2016).

**Publisher’s note:** Springer Nature remains neutral with regard to jurisdictional claims in published maps and institutional affiliations.

## Supplementary Material

Supplementary Information

## Figures and Tables

**Table 1:** The mass of PCB and PBDE congeners in egg and neonate chick (ng/egg or ng/tissue).

	N	Weight (g)	Lipid (%)	PCB 95	PCB 91	PCB 149	PCB 132	BDE 47	BDE 100	BDE 99	BDE 85	BDE 154	BDE 153	BDE 183	BDE 209
Background	3	43.6 ± 2.03	10.6 ± 0.75	2.84 ± 0.93	2.15 ± 0.89	1.27 ± 0.31	1.78 ± 0.38	0.32 ± 0.05	nd	nd	0.01 ± 0.01	0.02 ± 0.02	0.13 ± 0.01	0.27 ± 0.08	0.30 ± 0.02
Egg															
0 day	4	42.3 ± 2.03	9.3 ± 0.55	352 ± 15.1	523 ± 24.1	397 ± 24.4	414 ± 24.2	133 ± 0.89	128 ± 10.7	130 ± 7.2	134 ± 9.20	113 ± 15.0	145 ± 4.80	121 ± 10.5	327 ± 11.3
18 day	4	41.0 ± 2.58	8.1 ± 1.8	292 ± 21.1	495 ± 21.0	383 ± 17.6	380 ± 16.7	137 ± 4.40	124 ± 7.31	133 ± 3.31	129 ± 4.50	119 ± 8.92	143 ± 10.8	116 ± 9.03	313 ± 13.9
Chick															
Heart	6	0.23 ± 0.05	2.1 ± 0.90	1.02 ± 0.12	0.16 ± 0.20	2.32 ± 0.19	2.92 ± 0.17	0.05 ± 0.07	0.71 ± 0.08	0.17 ± 0.07	0.25 ± 0.12	0.18 ± 0.09	nd	nd	nd
Stomach	6	1.59 ± 0.34	1.7 ± 0.17	2.01 ± 0.42	2.31 ± 1.58	4.54 ± 1.63	5.07 ± 1.44	0.66 ± 0.17	1.65 ± 0.20	0.86 ± 0.16	0.72 ± 0.15	1.43 ± 0.32	0.31 ± 0.24	1.27 ± 0.24	3.71 ± 3.49
Liver	6	0.98 ± 0.14	11.5 ± 0.55	8.73 ± 1.34	37.9 ± 5.10	17.1 ± 3.10	24.5 ± 3.87	5.79 ± 1.11	11.2 ± 2.35	8.00 ± 1.24	4.18 ± 1.89	8.96 ± 3.27	15.3 ± 2.76	17.2 ± 2.90	70.9 ± 26.9
Carcass	6	22.2 ± 2.34	5.2 ± 1.3	79.5 ± 19.8	144 ± 39.6	149 ± 28.6	130 ± 27.1	87.3 ± 13.3	69.6 ± 10.9	68.8 ± 8.01	44.4 ± 9.30	60.6 ± 11.6	46.5 ± 5.17	60.0 ± 15.1	40.3 ± 5.33
Σ Chick	6	25.0 ± 2.67		91.3 ± 18.9	185 ± 39.0	172 ± 29.3	163 ± 26.4	93.8 ± 13.3	83.1 ± 12.3	77.8 ± 8.47	49.5 ± 10.5	71.2 ± 10.5	62.0 ± 5.75	78.5 ± 15.7	115 ± 28.5
Yolk	6	3.69 ± 1.56	22.2 ± 5.8	68.5 ± 23.5	128 ± 38.1	109 ± 24.4	118 ± 27.6	40.5 ± 9.82	45.0 ± 10.5	37.5 ± 7.83	34.3 ± 8.40	40.7 ± 12.0	40.8 ± 7.61	39.0 ± 11.8	139 ± 24.8
Shell	6	3.9 ± 0.47		0.63 ± 0.52	0.37 ± 0.28	nd	0.37 ± 0.29	1.1 ± 1.2	0.42 ± 0.39	0.47 ± 0.37	0.52 ± 0.44	1.5 ± 1.3	0.83 ± 0.59	1.0 ± 0.88.	47 ± 30

nd: Under detected limitation.
