# Supplementary material for: In ovo uptake, metabolism, and tissue-specific distribution of chiral PCBs and PBDEs in developing chicken embryos
Source: Sci Rep. 2016 Nov 7;6:36597. doi: 10.1038/srep36597 (PMC5098194; doi:10.1038/srep36597)
Supplement: Supplementary Information [file srep36597-s1.doc]

**Support information**

***In ovo* uptake, metabolism, and tissue-specific distribution of chiral PCBs and PBDEs in developing chicken embryos**

Zong-Rui Li^1,2^, Xiao-Jun Luo^1^,* Li-Qian Huang^1,2^, Bi-Xian Mai^1^

^1^State Key Laboratory of Organic Geochemistry and Guangdong Key Laboratory of Environmental Protection and Resources Utilization, Guangzhou Institute of Geochemistry, Chinese Academy of Sciences, Guangzhou 510640, China

^2^University of Chinese Academy of Sciences, Beijing 100049, China

^*^ Corresponding author. Tel.:86-20-85297622; fax: 86-20-85290706

*E-mail address*: [luoxiaoj@gig.ac.cn](mailto:luoxiaoj@gig.ac.cn) (X.-J. Luo)

**Sample Preparation.** Briefly, egg shell samples were extracted with 200 mL hexane/dichloromethane (1/1, v/v) for 48 h after being spiked with the surrogate standards (6-OH-BDE87). The extract was concentrated to 2 mL and partitioned with a total of 12 mL of potassium hydroxide (KOH, 0.5N in 50% ethanol). The KOH phase (the alkaline phase) used for OH-PBDEs analysis was acidified with hydrochloric acid to PH < 2, then were extracted with 12 mL hexane: MTBE (1:1, v/v) and dried by anhydrous Na_2_SO_4_. Samples were derivatized to their methoxy analogues using diazomethane. After methylation, the samples were purified on a silica gel column (i.d=1.0 cm) packed with 8 cm neutral silica (3% deactivated, w/w) and 8 cm sulfuric acid silica (56:44, w/w) topped with a 2 cm layer of anhydrous Na_2_SO_4_ and eluted with 30 mL of DCM : hexane (1:1, v/v). The extract was evaporated to near-dryness and redissolved in 200 μL iso-octane and internal standard (6-MeO-BDE85) was added before GC/MS analysis.

Figure Caption

Figure S1. Uptake efficiencies (%) of exposed chemicals (PCBs 91, 95, 149 and 132; PBDEs 47, 85, 99, 100, 154, 153, 183 and 209). Error bars represent standard deviations.


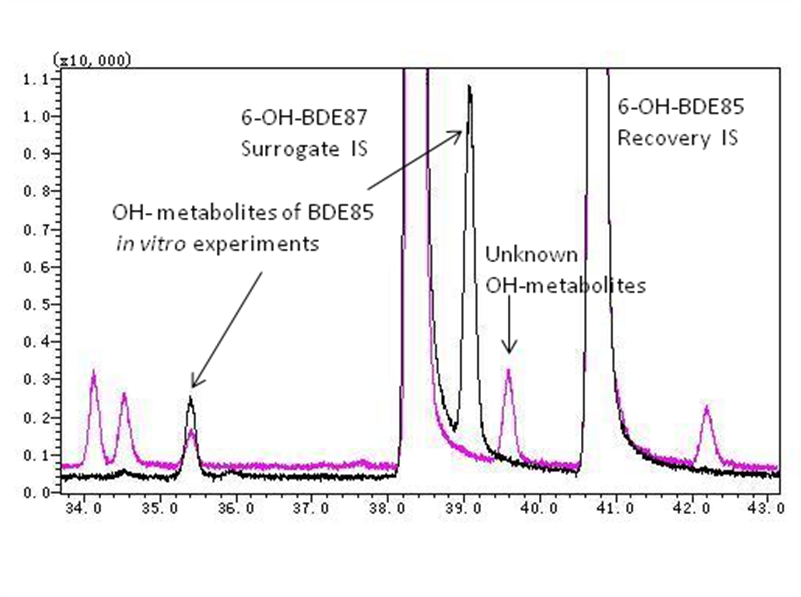


Figure S2. Typical selected ion mass chromatograms of brominates ion isotope (*m/z* 79 and 81) for *in vitro* experiment of BDE85 (the black line) using chicken liver microsome and OH-PBDEs (derivatized to MeO-PBDEs) in egg shell alkaline fraction of present *in vivo* study (the red line).

Figure S3. Correlation analysis between mean concentration ratio and the log *Kow* of chemicals (PCBs 91, 95, 149 and 132; PBDEs 47, 85, 99, 100, 154, 153, 183 and 209).
